# Factors associated with stunting: gut inflammation and child and maternal-related contributors among under-five children in Hawassa City, Sidama Region, Ethiopia

**DOI:** 10.1186/s40795-023-00701-4

**Published:** 2023-03-21

**Authors:** Berhanu Kibemo Lefebo, Dejene Hailu Kassa, Baye Gelaw Tarekegn

**Affiliations:** 1 School of Nutrition, Food Science and Technology, College of Agriculture, University of Hawassa, Hawassa, Ethiopia; 2 School of Public Health, College of Medicine and Health Sciences, Hawassa University, Hawassa, Ethiopia; 3 Department of Medical Microbiology, School of Biomedical and Laboratory Sciences, College of Medicine and Health Sciences, University of Gondar, Gondar, Ethiopia

**Keywords:** Bacterial pathogens, Children, Fecal leukocytes, Gut inflammation, Stunting, Undernutrition

## Abstract

**Background:**

Under-nutrition remains a major global public health challenge, particularly among children under the age of five. Among the manifestations of under-nutrition, stunting accounts for the larger proportion, which is associated with multiple factors. In Ethiopia, however, the link between intestinal inflammation and childhood stunting was not well investigated. Therefore, the present study aimed to determine the association between gut inflammation and childhood stunting.

**Method:**

A community-based cross-sectional study was conducted and a total of 82 children were included in the study. Anthropometric data were collected by measuring weight in underwear and without shoes with an electronic scale to the nearest 0.1 kg and their height in the Frankfort plane with a telescopic height instrument. Environmental risk factors for enteric bacterial exposure, access to improved sources of drinking water, and the presence of facilities for hygiene and sanitation conditions were assessed using a questionnaire. Gut inflammation was tested through fecal leukocyte count and each sample was stained with methylene blue. Stool samples were inoculated on MacConkey agar, Salmonella-Shigella agar, and Xylose Lysine Deoxycholate agar after enrichment with Selenite cystine broth and incubated at 37 °C for 18–24 h. Binary and multiple logistic regressions and Chi-square models were used to analyze the data.

**Result:**

Data from the current study revealed that gut inflammation was (AOR: 5.28, 95% CI: 1.32–22.25) associated with stunting. On the other hand, children with reported diarrhea within the last week were 6 times more likely for the probability of being stunted (AOR: 6.21, 95% CI: 2.68–26.83). The findings of this study also demonstrated that children from a household with a family size of more than 5 members were three times more likely to be stunted than their counterparts (AOR: 3.21, 95% CI: 1.20 -10.13). Facts of the current study demonstrated that breastfeeding for 24 months and below was negatively associated (AOR: 0.3; 95% CI: -0.46-0.89) with gut inflammation. Detection of *E.coli* and *Shigella* species in the stool samples of children and Menaheria residents were positively associated with gut inflammation (AOR: 5.4, 95% CI: 1.32–22.25; AOR: 5, 95% CI: 1.47–24.21), respectively.

**Conclusion:**

Therefore, there was a strong correlation between stunting and gastrointestinal inflammation. Moreover, stunting was associated with diarrhea, breastfeeding duration, residence, and family size. Similarly, intestinal inflammation was linked to residence, breastfeeding duration, and the prevalence of bacterial infections such as *E. coli* and *Shigella* species.

## Introduction

 Under-nutrition remains a major global public health challenge, particularly among children under the age of five[1]. It continues to be a major cause of morbidity and mortality, especially in developing countries [2]. Stunting is the most prevalent [3] form of under-nutrition that affected about 149 million under-five children at the global level[4]. Stunting accounts for 14–17% of mortality in under-five children worldwide. It leads to long-term cognitive declines, less time and less performance at school, reduced adult operating performance, and a greater risk of delay in descendants [5]. Childhood nutritional stunting might lead to an increased risk of infections and metabolic disorders, lower fat oxidation, higher risk of creating diabetes, adulthood obesity, and hypertension [6].

Many research findings demonstrated that factors that contribute to stunting are multifaceted [7]. It was linked to biological, behavioral, and environmental variables [8, 9]. According to previous study reports, the prevalence of stunting in the East African region is influenced by factors such as fertility rate, child nutrition, and feeding patterns [10, 11]. Among the environmental factors contributing to stunting, poor drinking water supply, sanitation, and hygiene (WaSH) situation are becoming more significant as it is one of the causes of gut inflammation [12, 13]. A healthy gut as a biological component is essential to achieving normal growth in children. Intestinal health is central to optimal growth and development because it maintains nutrient retention while preventing the body from excessive microbial introduction, illness, and systemic inflammation [14].

Infectious or non-infectious inflammation in maternal during pregnancy and neonates has an essential influence on young children’s growth as well as overall health. A growing literature has arisen with strong evidence that inflammation is negatively and causally associated with height for age among under-five children [15, 16]. Some other studies also have argued for an inverse association between inflammation and cognitive maturity rather than physical growth in children [17, 18]. Gut inflammation, one of the implications of environmental enteropathy, is affecting children’s nutritional status through malabsorption of essential nutrients, and activation of the immune system at expense of cellular proteins and carbohydrates [19]. The health of the gut mucosa is dependent on the composition and metabolic activity of the microbial communities that live there [20]. Preventing the entry of enteric pathogens and other pathogenic microbes through improved WaSH services could prevent the majority of gut disorders in developing countries [12].

Although the association between gut inflammation and childhood stunting is reported elsewhere in previous studies [21, 22], the findings lack consistency because of differences in study designs and biomarkers used to test gut inflammation [14, 23, 24]. However, in Ethiopia, studies have indicated that WaSH status is associated with childhood stunting but have not addressed the concurrent link with gut inflammation. Therefore, the aim of the present study was to determine the association between gut inflammation and childhood stunting in Hawassa City, Sidama Regional State, Ethiopia.

## Materials and methods

### Study area and period:

This study was performed in Hawassa City, the capital city of the Southern Nation, Nationalities and Peoples Region (SNNPR), and Sidama National Regional State. Hawassa is located at 70’ 03” latitude, 80’29” east longitude, at 7° 3’ 0” N latitude, 38° 28’ 0” E longitude. The City is 270 km South of Addis Ababa, the capital city of Ethiopia. Hawassa is bordered by the Wondo Genet District in the East, the South Dore Bafeno District in the South, the Oromia Region in the North, and the Hawassa Lake and Oromia Region in the West. The metropolis is divided into 8 sub-cities and 32 kebeles. According to the housing and population census, the projected population of Hawassa metropolis administration in 2011 E.C. was 329, 734, out of which 169, 677 were males and 160, 057 were females [25]. However, the actual total number of under-five children in the city in 2021 was 27,651, based on the statistical data of Hawassa City’s health bureau. The study was conducted from July 2021 to August 2021.

### Source population, study population, and study design:

The source population for this study was all children aged under five years, and living in Hawassa City. Children aged 6–59 months and stunted ones were eligible for this study. The data were collected using a community-based cross-sectional study design.

### Sample size determination:

The sample size was determined using G-power by considering the Chi-square test, α value of 0.05, β value of 0.20, estimated effect size of 0.3, and degree of freedom to be 1 [26]. We assumed a medium effect size based on the close and substantial correlation between environmental enteric dysfunction or gut inflammation and growth faltering in children in developing countries [13, 27]. Thus, the total sample size was calculated and determined to be 88. However, data from 82 child-mother pairs were used for the analysis.

### Sampling technique:

Primary sampling unit was the sub-city in Hawassa City, and then the kebeles and the households in the kebeles were the secondary and tertiary sampling units respectively. Eventually, a child-mother pair was selected as a quaternary sampling unit among the source population through simple random sampling. After having the total number of under five children in the city, different numbers of under-five children were selected for each sub-cities based on the city’s demographic data in 2021 through probability proportional to size technique. A household was eligible to participate in the study if it had under-five children and was willing to participate.

### Inclusion and exclusion criteria:

Children with age 6–59 months, beginning of solid food intake, and stunted growth were used as inclusion criteria, and those fulfilling these criteria were eligible for the study. Children with any of the exclusion criteria below were not eligible for the study: not exclusively breastfeeding, any chronic illness and any malnutrition case other than stunting.

### Data collection tools and procedures:

For anthropometric data collection from children, permission was obtained from the Parents/guardians. The kebeles’ health extension workers participated in the data collection. Data were collected from children aged 24–59 months by measuring their weight in underwear and without shoes with an electronic scale (Type SECA 861 or SECA 813, Hamburg, Germany) to the nearest 0.1 kg and their height in the Frankfort plane with a telescopic height instrument (Type SECA 225 or SECA 214) to the nearest 0.1 cm. Data from children aged 6–23 months, on the other hand, were collected by measuring the child’s weight and that of the mother/guardians, then subtracting the weight of the mother/guardians from the sum weight of the child and the mother/guardians. The height was measured using an accurately graduated length board and recorded to the nearest millimeter. The age of the children was obtained from a parental recall using an events calendar. The height and the weight of the children were measured twice, and the average was taken. The measuring instruments were calibrated at least twice a day in each case. Nutritional status indices were generated by using WHO Anthro software for the children. According to the World Health Organization (WHO), wasting, stunting, and being underweight are defined as Z-scores of less than − 2 standard deviations of weight for height, height for age, and weight for age, respectively[28]. To assess the environmental risk factors for enteric bacterial exposure of the children, the children’s household environment was determined by the data collectors following the guidelines [29] for access to improved sources of drinking water, the presence of facilities for hygiene, and sanitation conditions.

### Gut inflammation test:

This was tested through fecal leukocytes count. Fresh stool samples that were collected by using sterile cups were examined for the presence of fecal leukocytes on smears made onto glass slides within 20–30 min after the stools were collected as described in clinical diagnosis guidelines[30, 31]. The stools for microscopic examination were chosen from an area with blood or mucus if present. Each sample was stained with methylene blue (Himedia, Mumbai, India) and examined by an experienced laboratory technician who was blinded to the source of the sample. Microscopic examination of the preparations was done by examining each for 10 min using an optical light microscope. The numbers of leukocytes per field (Lpf) (Oil immersion field, magnification, 1000x) were determined in at least 20 fields. The average results were categorized as follows: 3 to 5 Lpf, 6 to 10 Lpf, 11 to 15 Lpf, or $\ge$ 16 Lpf. Based on previous studies, we chose a cutoff point of 10 Lpf to decide the presence of a gut inflammation related to an enteric infectious bacteria [32].

### Bacterial culture as a conformation test:

Stool samples were collected using clean, dry, and leak-proof stool cups and immediately placed into the Cary-Blair transport medium (Oxoid Ltd., Basingstoke, UK). Samples were transported to Hawassa University’s Food Microbiology Laboratory in cold boxes with ice packs within 2 h of collection for further processing. Stool samples were directly inoculated onto MacConkey agar, Salmonella-Shigella agar, and Xylose Lysine Deoxycholate agar after enrichment with Selenite cystine broth and incubated at 37 °C for 18–24 h. After incubation, bacterial isolates were identified to the genus level by the colony morphology and biochemical characteristics of the isolates[33].

### Data analysis

Before the data processing, filled questionnaire was checked for completeness and consistency. Then, the data were coded, entered, and cleaned using SPSS version 20⋅0 (SPSS, Inc., Chicago, IL, USA). Data were analyzed by using both descriptive as well as the inferential statistics. Assumptions for the statistical models used for data analysis were checked and fulfilled. Eventually, the data were analyzed by using Chi-square, and bivariate and multivariate logistic regression to identify significant associations at p < 0.25 and p < 0.05 respectively.

## Results

### Characteristics of the study subjects

Socio-demographic characteristics of the study subjects showed that the majority (54.9%) of them were within the age of 13–35 months followed by 36–59 months (32.9%). On the other hand, 53.7% of them were male and 95.1% of their family were married but the other 4.9% were divorced. The nutritional status of the children showed that 41.5% of them were stunted. Clinical characteristics of the children showed that 23.2% had diarrhea within the last one week. Data on the immunization status of children showed that 94% of them were immunized. On the other hand, 36.6% of the children enrolled in the current study were exposed to antimicrobial agents within the last 3 months. The macroscopic examination of the stool samples showed that 28% had mucus, 3.7% had blood and 57.3% had formed stool samples (Table 1).

**Table 1:** Socio-demographic and clinical characteristics of children aged 6–59 months at Hawassa City, Sidama Region, Ethiopia, 2022

Characteristics	Category	Frequency	Percentage
Age in month	6–12	10	12.2
	13–35	45	54.9
	36–59	27	32.9
Sex	Male	44	53.7
	Female	38	46.3
Household’s marital status	Married	78	95.1
	Divorced	4	4.9
Household head	Male	64	78
	Female	9	11
	Both	9	11
Family size	< 5	9 38	11 46.3
	≥ 5	44	53.7
Nutritional status	Stunted	34	41.5
	Not stunted	48	58.5
Diarrhea in the last week	Yes	19	23.2
	No	63	76.8
Immunization status	Complete	77	94
	Incomplete	5	6
Antibiotic exposure in the last 3 months	Yes	30	36.6
	No	52	63.4
Duration of breastfeeding in months	≤ 24	26	31.7
	> 24	56	68.3
Presence of mucus on a stool sample	Yes	23	28
	No	59	72
Consistency of stool sample	Formed	47	57.32
	Not formed	35	42.68
Presence of blood on a stool sample	Yes	3	3.66
	No	79	96.34
Mother’s education status	Illiterate	15	18.3
	Grade − 8	40	48.8
	≥9 grade	27	32.9

### The association between gut inflammation and stunting

**Fig. 1:**
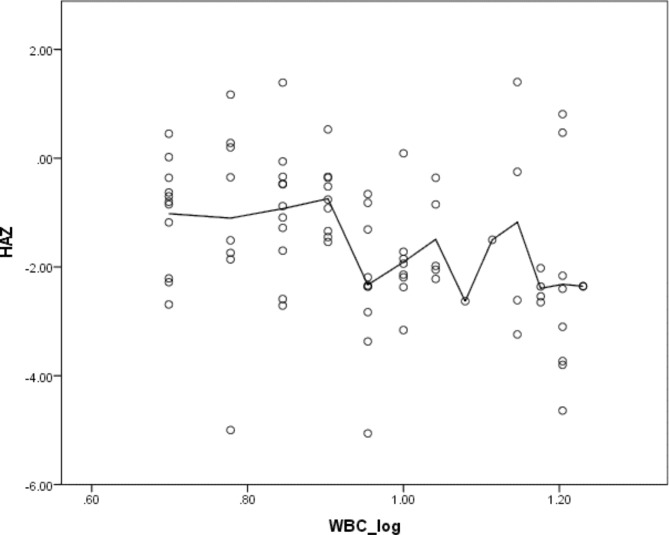
Correlation between log-transformed WBC count and HAZ among under-five children at Hawassa City, Sidama Region, Ethiopia

A Pearson’s correlation coefficient was computed to assess the relationship between gut inflammation and height for age (HAZ) among under-five children. According to the results of the analysis, gut inflammation was negatively correlated (r = − 0.323, n = 82, *p* = 0.003) with HAZ. Overall, there was a weak and negative correlation between gut inflammation and the children’s HAZ. Increases in gut inflammation were correlated with decreases in HAZ as demonstrated in the scatter plot (Fig. 1).

Multivariate regression analysis of the current data demonstrated that children who had gut inflammation were 5.28 times more likely to be stunted than their counterparts (AOR: 5.28, 95% CI: 1.61–20.21). On the other hand, children with reported diarrhea within the last one week before the stool sample collection was 6.21 times increased probability of being stunted (AOR: 6.21, 95% CI: 2.68–26.83). Surprisingly, children who breastfed for more than 24 months were more likely to be stunted as compared with those who breastfed for less or equal to 24 months (AOR: 1.23, 95% CI: 1.09–11.90). Children belonging to a household with a family size of 5 and more were 3.21 times more likely to develop stunted than their counterparts (AOR: 3.21, 95% CI: 1.20 -10.13). Children who lived in the Haik Dar sub-city of Hawassa city had a 1.61 times greater chance of being stunted than Tulla sub-city (AOR: 1.61, 95% CI: 1.12–17.34). When compared to the reference age group (6–12 months), stunting was associated with children’s ages 13–35 months, but this link was not statistically significant (Table 2).

**Table 2:** Determinants of stunting among under-five children at Hawassa City, Sidama Region, Ethiopia, 2022

Variables	Category	AOR	95% C.I.	*p*
Antibiotics exposure	Antibiotics exposed	1.81	0.44-7.38	0.421
Duration of breastfeeding in months	> 24 months	1.23	1.09–11.90	0.043*
Hygiene facility	Hand washing w/out soap	2.14	0.88 − 29	0.710
Improved drinking water source	Not available in the yard	3.37	0.29 − 21	0.342
Sex	Male	0.47	0.11-2.19	0.334
Age_cat in moths	13–35	4.09	0.54-18.90	0.170
Immunization	Not complete	3.7	0.12-15.60	0.351
Residence	Haik Dar sub-city	1.61	1.12–17.34	0.032*
WBC_cat	Inflamed	5.28	1.61–20.21	0.007*
Family size	≥5	3.21	1.20-10.13	0.041*
Diarrhea in the last week	Present	6.21	2.68–26.83	0.002^*^

p^*^: significant at *p* < 0.05

### Factors associated with gut inflammation among children

The current study revealed that breastfeeding for 24 months and below was negatively associated (AOR: 0.3, 95% CI: -0.46-0.89) with gut inflammation in the children. However, the prevalence of enteric bacteria (*E.coli and Shigella*) in the stool and living in the Menaheria sub-city were positively associated (AOR: 5.4, 95% CI: 1.32–22.25; AOR: 5.0, 95% CI: 1.47–24.20) with gut inflammation respectively. The rest three variables: drinking water supply (AOR: 3.3, 95% CI: 0.7-15.88), reported diarrhea in the last week (AOR: 1.39, 95% CI: 0.82–2.83), and the presence of mucus in the stool (AOR: 0.5, 95% CI: 0.13–1.92) were not statistically significantly associated with gut inflammation (Table 3).

**Table 3:** Factors associated with gut inflammation among under-five children at Hawassa City, Sidama region, Ethiopia, 2022

Variables	Category	p	COR	Lower	Upper	p	AOR	Lower	Upper
Duration of breastfeeding	≥24months	0.46	1.4	0.5	3.7	0.048*	0.302	-0.462	0.897
Improved drinking water source	Not present	0.5	0.6	0.2	2.3	0.129	3.346	0.705	15.885
Bacteria detected in the stool	*E.coli* and *Shigella*	0.27	0.73	0.4	1.3	0.019*	5.424	1.321	22.259
Diarrhea in the last week	Present	0.25	1.8	0.6	5.1	0.231	1.389	0.821	2.838
Mucus in the stool	Present	0.2	1.93	0.72	5.2	0.313	0.501	0.131	1.924
Residence of the child	Menaheria	0.94	0.9	0.25	3.6	0.021*	5.034	1.47	24.209
	Tabor	0.065	0.13	0.01	1.14	0.163	3.507	0.621	18.538
	Haik Dar	0.568	0.74	0.23	2.2	0.23	2.612	0.574	12.136

p^*^- statistically significant at *p* < 0.05

## Discussion

The purpose of the current study was to determine whether gut inflammation is linked with childhood stunting and other factors associated with gut inflammation. Our findings revealed that gut inflammation is associated with childhood stunting among the sampled population. This is supported by previous research reports that gut inflammation is an indicator of gut damage that affects the body’s nutrient acquisition [34–36], thereby contributing to stunting. Similar to the current report, another cohort follow-up study pointed out that childhood growth faltering is strongly associated with systemic inflammation, and caused by gut damage [37]. On the contrary to prospective study among Bolivian infants reported no association between inflammation and stunting[38]. This would probably be due to the infancy age of the study population as the age of children is positively related to stunting[39].

The current study demonstrated that some risk factors such as the presence of diarrhea in the past week, large family size, residence, and breastfeeding duration of more than 24 months showed a statistically significant association with childhood stunting in addition to gut inflammation. Many previous reports in Ethiopia also documented that diarrheal frequency, as well as the duration as the predictor of childhood stunting [40–42], which might be a result of repeated infections and leads to essential micronutrient loss, reduced appetite, and an increase in the demand for metabolites of biosynthesis.

Similar to our results, a family size of 5 or more has been determined to be a risk factor for stunting in rural areas of Ethiopia and elsewhere. The possible explanation for this would be the negative association between family size and the households’ food security and less attention for under-five children in the household. Prominent explorations have addressed the positive importance of breastfeeding for optimum growth in children. Those studies stressed only the nutritional value of breast milk for children during their infancy age, but not a change in the composition of breast milk through time. However, our study observed that the extension of the duration of breastfeeding above 24 months decreased the children’s height for age. Inconsistent with our findings, a study from Pakistan implied that children’s nutritional status changes from stunting to severe stunting as the duration of breastfeeding increases after the second year of life [43]. The contributing linkage of longer breastfeeding duration with stunting would likely be a result of the fatty composition of breast milk that is associated with gut inflammation [44, 45], and low appetite for complementary foods that promote linear growth in children.

Data of the current study demonstrated that gut inflammation is significantly associated with the prevalence of enteric bacteria in stool, duration of breastfeeding up to 24 months, and the residence of the children. Many studies have found a link between intestinal inflammation and bacterial infections. A longitudinal study conducted on a birth cohort came up with a direct relationship between gut inflammation and exposure to pathogens’ invasion in early life. The study has also indicated a negative association between gut inflammation and length for age Z-scores[36]. An observational study carried out in the different environments of the same town in India showed that the odds of gut inflammation increase with the prevalence of intestinal pathogens associated with poor WaSH situations [46]. However, we did not see a significant association between WaSH conditions and enteric bacterial pathogens’ prevalence in the present study. The inconsistency between the findings might be explained by the larger sample size, diversity of the groups of gut pathogens identified, and methodological difference in the case of the Indian report. Another study in rural Ethiopia tested that improved drinking water supply decreased the chance of getting gut inflammation among under-five children [47], which is unlike our findings that did not show a significant association. The disparity could be due to a difference in the settings (rural/urban) of the study population.

Based on the findings of this study, the duration of breastfeeding up to 24 months was found to be protective against gut inflammation. This is in agreement with various investigations conducted in middle and low-income countries that established the role of breastfeeding in keeping up the gut health in children [48–50]. The fact would probably be related to the healthy proportional composition of breast milk during the first 2 years, and then after that tends to have more fat components than the other macronutrients. According to Imhann F et al., a diet with a high content of fat and sugar increased the concentration of fecal calprotectin in the stool as a biomarker of gut inflammation. The study has also suggested that high fat and sugar in a diet aggravate gut inflammation via the promotion of the growth of pro-inflammatory enteric bacteria[51, 52]. Our findings also revealed that the living of the children in the Menaheria sub-city was positively related to gut inflammation. This could be linked with the contribution of a living environment to the exposure of biological or chemical factors triggering gut inflammation. Likewise, studies carried out under different environmental settings have documented that a certain surrounding has either a protective or predisposing effect on gut inflammation [53, 54].

## Conclusion

Data from the current study showed that gut inflammation is significantly associated with stunting among children. Besides gut inflammation, family size, diarrhea in the last week, the living residence, and breastfeeding duration for longer than 24 months were predictors of stunting in children. Gut inflammation was also associated with the prevalence of *E.coli* and *Shigella* species in the stool, breastfeeding duration, and the living residence. Accordingly, we strongly recommend that the government’s public health policy should give priority to the awareness creation at both the households and community level about family planning, breastfeeding duration, and environmental hygiene.

## Data Availability

All data generated or analyzed during this study are included in this published article.
